# High Temperatures Are Required for Expression of Metabolic Resistance to Cyhalofop-Butyl in a Biotype of *Echinochloa crus-galli*

**DOI:** 10.1021/acs.jafc.5c16988

**Published:** 2026-04-03

**Authors:** Luan Cutti, André Lucas Simões Araujo, Guilherme Menegol Turra, Carlos Alberto Gonsiorkiewicz Rigon, Paula Sinigaglia Angonese, Mateus Gallon, Franck E. Dayan, Todd A. Gaines, Aldo Merotto

**Affiliations:** † Department of Crop Science, 28124 Federal University of Rio Grande do Sul, Porto Alegre, Rio Grande do Sul 91540-000, Brazil; ‡ Department of Agricultural Biology, 3447 Colorado State University, Fort Collins, Colorado 80523, United States

**Keywords:** ACCase inhibitor, barnyardgrass, cytochrome P450, climate change

## Abstract

Rising average global temperatures due to climate change may affect weed survival to herbicides. The metabolic resistance of *Echinochloa crus-galli* to cyhalofop-butyl was characterized at 25/20 °C and 35/30 °C. The biotype SAOJER-R was resistant at 35/30 °C with GR_50_ > 525 g ai ha^–1^ but was susceptible at 25/20 °C with GR_50_ < 52 g ai ha^–1^. SAOJER-R biotype needed at least 48 h of exposure to 35/30 °C after application to express the resistant phenotype. The resistant biotype metabolizes cyhalofop-butyl and acid faster than the susceptible, and faster at 35/30 °C than at 25/20 °C. The application of a cytochrome P450 monooxygenase inhibitor with cyhalofop-butyl increased the amount of active herbicide recovered in plants at 25/20 °C. Seven *cytochrome P450 monooxygenase*, seven *glutathione-S-transferase*, and five *UDP-glycosyltransferase* genes were upregulated. Temperature is critical for expression of SAOJER-R metabolic resistance to cyhalofop-butyl at label rate. We suggest that temperature conditions should be standardized when evaluating metabolic herbicide resistance.

## Graphical abstract

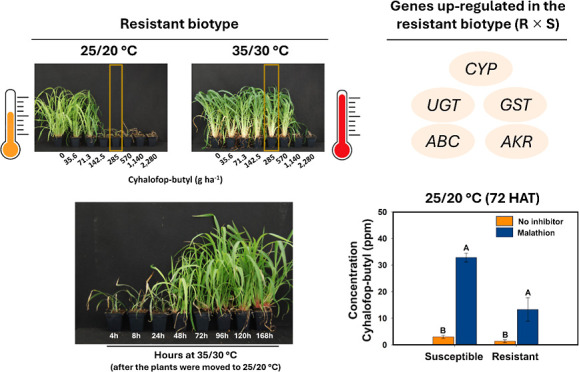

## 1 Introduction

*Echinochloa* spp. are major weeds in rice fields and have recently invaded corn and soybean fields. Several herbicide modes of action are effective in controlling this genus; however, many populations have been reported to be resistant to most modes of action, such as acetolactate synthase (ALS) inhibitors, auxin mimics (florpyrauxifen-benzyl and quinclorac), glyphosate, photosystem II inhibitors, very long chain fatty acid inhibitors, and acetyl-CoA carboxylase (ACCase) inhibitors.^1^ The ACCase inhibitors became the main postemergence chemical control mode of action in rice due to widespread of quinclorac^2^ and ALS-resistant *Echinochloa* populations.^3^ The ACCase mode of action is well-known to control only grass species; however, some specific active ingredients are selective in rice, such as the aryloxyphenoxypropionate chemical group members cyhalofop-butyl and fenoxaprop-*p*-ethyl.^4,5^ The selectivity of the pro-herbicide cyhalofop-butyl in rice is due to the reduced conversion rate to the active form cyhalofop acid followed by an increased metabolism into nontoxic metabolites,^4^ while fenoxaprop-*p*-ethyl selectivity relies on use of the safener isoxadifen-ethyl that induces formation of inactivated herbicide conjugates.^6^ The metabolic selectivity of ACCase-inhibitor herbicides in rice makes them candidates to also evolve metabolic resistance in grass weeds, especially in barnyardgrass (*Echinochloa crus-galli*) due to its genomic architecture, an allohexaploid species with a high number of genes potentially involved in herbicide metabolism (867 *CYP* (*cytochrome P450 monooxygenase*), 227 *GST* (*glutathione-S-transferase*), and 361 *ABC* (*ATP-binding cassette*) genes).^7^ These gene families are also related to herbicide resistance in *Echinochloa* spp. Genes from *CYP*, *GST*, *ABC*, and *AKR* (*aldo-keto reductase*) families were associated with resistance to penoxsulam and bensulfuron-methyl,^8^ florpyrauxifen-benzyl,^9^ glyphosate,^10,11^ and ACCase inhibitors^12^ in *Echinochloa* *.*

Both target-site mutations and metabolic resistance have been reported in *Echinochloa* populations resistant to ACCase inhibitors. The Asp2078Glu,^13^ Trp1999Cys, Trp2027Cys/Ser, and Ile2041Asn^14^ mutations in the *ACCase* gene confer resistance to cyhalofop-butyl in *E. crus-galli*. This species is an allohexaploid species (2*n* = 6*x* = 54) and has five *ACCase* genes located in Chr 9A, 9B, 9C, 5B, and 5C, and one pseudogene located in Chr 5A, but a mutation in only one out of five functional genes is sufficient to confer the resistant phenotype.^14^ On the metabolic side, the metabolism of ACCase inhibitors in several weed species is mainly related to CYP450 or GST enzyme pathways.^15^ In *E. crus-galli*, the utilization of fenoxaprop-*p*-ethyl with the safener isoxadifen-ethyl selected a biotype with fenoxaprop-*P*-ethyl resistance induced by the safener, probably inducing metabolism driven by GST enzymes, while remaining susceptible to cyhalofop-butyl and other ACCase inhibitors.^16^ The genes *CYP81A68* ^17^ and *GSTU23* ^12^ were validated through heterologous expression, along with three *UGT (UDP-glycosyltransferase)* candidate genes not validated,^18^ which have been reported to metabolize cyhalofop-butyl in *E. crus-galli*. The *CYP709C69* gene from *Echinochloa phyllopogon* was associated with diclofop-methyl resistance but not with cyhalofop-butyl, when transformed into yeast and rice *calli*.^19^ *CYP81A12* and *CYP81A21* from *E. phyllopogon* with diclofop-methyl, tralkoxydim, and pinoxaden resistance were validated through heterologous expression in transformed rice.^20^ One additional ACCase resistance mechanism has been reported in *Digitaria sanguinalis*, and it refers to an increased number of *ACCase* genes in the resistant population compared with two gene copies in susceptible populations.^21^

Herbicide resistance is responsive to temperature, especially when related to metabolic resistance. Increasing the temperature generally makes plants less sensitive to herbicide application, likely due to a general increase in enzymatic activity, including in susceptible plants.^22–25^ Temperature is one of the main environmental factors that has been increasing with the progress of global warming,^26^ and this may favor the evolution of herbicide-resistant weed populations. The recurrent selection of a susceptible *Echinochloa colona* population with subdoses of herbicide combined with heat stress across three seed generations decreased the sensitivity to florpyrauxifen-benzyl, imazethapyr, quinclorac, and glufosinate-ammonium.^27^ Rice is cultivated during the summer, and weed species found in rice fields are commonly exposed to high temperatures during long periods. The objective of this study was to investigate the resistance mechanism of an *E. crus-galli* population to cyhalofop-butyl and its interaction with temperature on the resistance level.

## 2 Materials and Methods

A bulk of seeds from several *E. crus-galli* plants surviving cyhalofop-butyl in a rice field, located in São Jerônimo, Rio Grande do Sul State, Brazil, were collected and screened in the greenhouse (G0) (28 °C ± 3 °C). The plants were sprayed with the label rate of 285 g ai ha^–1^ cyhalofop-butyl (Clincher, 180 g L^–1^, Corteva Agriscience, Indianapolis, USA) plus 1.5 L ha^–1^ of the vegetable oil Veget’Oil (930 g L^–1^, Oxiquímica Agrosciência, Jaboticabal) adjuvant, at 200 L ha^–1^ of spray volume. Survivors were selected to produce seeds (G1). A second screening was performed with G1 also in the greenhouse but in winter (20 ± 3 °C). There was a drastic variation in the progeny (G1) screening compared to the field seeds (G0) to the same herbicide and dose, where all G1 plants were killed. The screening with the same G1 progeny was repeated under winter conditions, and again the plants did not survive. The next procedure was screening the plants at 35 °C in a growth chamber, and in this condition cyhalofop-butyl resistance was observed. The screening was repeated at high temperature in a growth chamber until the seeds of the fourth generation (G4) were obtained and utilized to perform the following experiments. The biotype was named SAOJER-R.

### 2.1 Dose–Response Curves in Different Temperatures

The cyhalofop-butyl *E. crus-galli*-resistant biotype SAOJER-R (also resistant to ALS inhibitors due to Trp574Leu mutation and increased metabolism^3^) and one susceptible, VALE VERDE-S (also susceptible to ALS inhibitors), were utilized to perform cyhalofop-butyl dose–response curves in growth chambers under two different temperatures. Factor A was the two biotypes. Factor B was eight doses of cyhalofop-butyl, 0, 4.4, 8.9, 17.8, 35.6, 71.3, 142.5, and 285 g ai ha^–1^ of cyhalofop-butyl (Clincher CA, 285 g L^–1^, Corteva Agriscience, Indianapolis) for the susceptible, and 0, 35.62, 71.25, 142.5, 285, 570, 1140, 2280 g ai ha^–1^ for the resistant biotype, plus 2.5% v/v of methylated seed oils (MSO) adjuvant. Factor C was the two temperature conditions, 25/20 °C or 35/30 °C day/night. The plants were sprayed at the three-to four-leaf stage using an automated spray chamber (Generation III, Devries Manufacturing) at a spray volume of 200 L ha^–1^. The soil was kept flooded during the entire experiment. The growth chamber conditions were 14 h/10 h light/dark, 400 μmol m^–2^ s^–1^ of light intensity, and humidity 70–80%. Each treatment had six replicates, and the entire experiment was carried out twice. The dry biomass was assessed 28 days after treatment (DAT). The data were submitted to analysis of variation (ANOVA), and the averages were fitted to the three-parameter log-logistic nonlinear regression model using the *drc* package in *R* as follows

$$y=\frac{d}{{\left(1+\frac{x}{e}\right)}^{b}}$$

where *y* is the dry biomass, *x* is the herbicide dose, *b* is the curve slope at the inflection point, *d* is the upper limit, and *e* is the inflection point, representing the dose that reduced 50% of the dry biomass (GR_50_ parameter). The GR_50_ was utilized to compare resistance levels between biotypes and temperatures.

### 2.2 *ACCase* Gene Sequencing

DNA from SAOJER-R and VALE VERDE-S plants was extracted using the CTAB method.^28^ Three primer pairs were used to amplify the carboxyl-transferase domain of five *ACCase* genes which contain the following known positions to confer resistance to ACCase-inhibitors: Ile1781, Trp1999, Trp2027, Ile2041, Asp2078, Cys2088, and Gly2096 (Table 1). The five *ACCase* gene sequences from *E. crus-galli* genome with the primer locations are described in Figure S1. Polymerase chain reactions (PCRs) were prepared for a final concentration of 1× PCR buffer, 2 mM MgCl_2_, 0.2 mM dNTP, 0.5 U *Taq* DNA polymerase (Invitrogen), 0.4 μM each primer, 50 ng μL^–1^ genomic DNA, and as much as needed DNase free water to complete 20 μL. The PCR was carried out in a thermocycler at 95 °C for 3 min; 40 cycles of 95 °C for 30 s, 60 °C for 45 s, and 72 °C for 60 s; 72 °C for 10 min. The fragment size of each primer pair was confirmed in agarose gel (2%). The PCR product was purified using ExoSAP-IT (Applied Biosystems) protocol before Sanger sequencing.

**Table 1:** Primers Targeting Partially the Carboxyl-Transferase Domain of *E. crus-galli* *ACCase* Genes, Covering All the Positions Known to Confer Resistance to Herbicides

primer name	primer sequence 5′–3′	annealing temperature	fragment size	known mutation positions covered by the primers	reference
ACCase1F	CTGCAGCTGGATAGTGGTGA	60 °C	195 bp	Ile1781	^16^
ACCase1R	ACCAAGCCGAGCAAGATAAG				
ACCase3F	CATAGCTGTGGAGACGCAAA	60 °C	245 bp	Trp1999, Trp2027, Ile2041	^16^
ACCase3R	ATTGTTGACCCAGCCTGAAG				
ACCase7F	CATTCCTATGGCTGGAGAGC	60 °C	233 bp	Asp2078, Cys2088, Gly2096	^16^
ACCase7R	CATTTCCAACCTTTGCACCT				

### 2.3 Different Time Exposures to Each Temperature and Their Effect on the Resistance Phenotype

Two sets of resistant biotype SAOJER-R plants were grown in two growth chambers at 25/20 °C or 35/30 °C day/night. At the three-to four-leaf stage, the plants were sprayed with cyhalofop-butyl at 285 g ha^–1^ plus MSO at 2.5% v/v, following the experimental procedures described above, and returned to their respective temperature. After that, plants were switched to the other temperature at 4, 8, 24, 48, 72, 96, 120, and 168 h after treatment (HAT). An additional set of untreated and treated plants was maintained at the same temperature (672 h). Each treatment had four replicates, with two plants per replicate. The experiment was carried out twice. The fresh biomass was assessed at 28 DAT. The data were fitted to the three-parameter log-logistic nonlinear regression model, described above, where in this case the parameter *e* represents the duration in hours at a specific temperature to reduce 50% of the fresh biomass (time_50_ parameter).

### 2.4 Quantification of Cyhalofop-Butyl and Cyhalofop Acid in Plants

The two *E. crus-galli* biotypes SAOJER-R, resistant, VALE VERDE-S, susceptible, were used to analyze the amount of cyhalofop-butyl (parent compound–molecular weight 357.14) and cyhalofop acid (active compound–molecular weight 301.08) by liquid chromatography–mass spectrometry (LC–MS)/MS. The experiment was conducted in two different temperatures, at 25/20 °C and 35/30 °C day/night, and the plants were grown and sprayed as described previously at 285 g ha^–1^ of cyhalofop-butyl plus MSO at 2.5% v/v. The plants were sampled at 8, 24, 72, 120, and 168 HAT. The CYP450 enzyme inhibitor malathion was sprayed at 1500 g ha^–1^ on additional plants 2 h before herbicide application, and those samples were collected at 72 and 120 HAT. Each treatment had four replicates, and each replicate consisted of two plants.

The aboveground biomass was collected, weighed, and washed 3× in different pots with 50/50% ethanol/water. The plants were placed in 50 mL conical tubes and ground using liquid nitrogen and pestle. Immediately, acetonitrile plus 0.1% formic acid was added at 2× mass/volume. A mechanical homogenizer was used to homogenize the samples for 50 s. A QuEChERS extraction kit was added to each sample and vortexed. The tubes were centrifuged at 3803 g for 20 min at 15 °C. The supernatant was collected and filtered using hydrophobic PTFE syringe filters, pore size 0.22 μm, and diameter 13 mm, into the vials. The LC–MS/MS system consisted of a Nexera X2 UPLC with 2 LC-30AD pumps, a SIL-30AC MP autosampler, a DGU-20A5 Prominence degasser, a CTO-30A column oven, and SPD-M30A diode array detector coupled to an 8040-quadrupole mass-spectrometer. Cyhalofop-butyl and cyhalofop acid were detected using the settings shown in Table S1.

The samples were chromatographed on a 100 × 4.6 mm Phenomenex Kinetex 2.6 μm biphenyl column maintained at 40 °C. Solvent A consisted of water with 0.1% formic acid and solvent B was MeOH with 0.1% formic acid. The solvent gradient was: 0 min 70% solvent B; 8 min 100% solvent B; 12 min 100% solvent B; 12.1 min 70% solvent B; 15 min 70% solvent B. The flow rate was set at 0.4 mL min^–1^, and each sample was analyzed as 5 μL injection volume. Calibration curves were made using 3, 10, 30, 100, 300, and 1000 pg of cyhalofop-butyl and cyhalofop acid technical grade standards, with *R* ^2^ of 0.999 and 0.999, respectively. The data were analyzed by ANOVA after checking assumptions for the analysis. The means at each time point between susceptible and resistant biotypes and the means of the same biotype at different temperatures were compared through the *t-test* (α = 0.05).

### 2.5 Differentially Expressed Genes in RNA-seq Analysis

The two *E. crus-galli* biotypes SAOJER-R (resistant) and VALE VERDE-S (susceptible) were grown in a growth chamber at 35/30 °C as described before. The plants were sprayed at 285 g ha^–1^ of cyhalofop-butyl at the three-to four-leaf stage, at a spray volume of 200 L ha^–1^. A segment of the two youngest leaves was collected at 3 HAT for the resistant and susceptible biotypes, plus untreated samples. Each treatment had three replicates, and each replicate consisted of two plants. The total RNA was extracted with Direct-zol RNA Miniprep Plus (Zymo Research) following the manufacturer’s protocol. During the extraction protocol, the samples were treated with DNase I to remove potential genomic DNA contamination. The RNA was sequenced at Novogene (California, USA) using Ilumina Novaseq 6000 paired-end 150 bp (6 GB raw data per sample).

The raw data were processed for adapter trimming and quality filtering using *fastp v. 0.23.2* package.^29^ The processed reads were aligned to the last version of *E. crus-galli* genome available^7^ using the package *HISAT2 v. 2.2.1*.^30^ The alignment files from *HISAT2* and the genome annotation file were used to count the mapped reads using the *featureCounts* command from *Subread v. 2.0.1* package.^31^ The analysis of read counts per contig and differentially expressed genes (DEGs) was done with the package *DESeq2* ^32^ in software R. In *DESeq2* package, a minimal prefiltering was performed to keep only contigs that have at least 10 reads total. A two-fold upregulated or downregulated genes (log2 fold change (LFC) of 1 and −1) cutoff was used, and a false-discovery rate (FDR) alpha = 0.05 (adjusted *p*-value). The contrasts of interest built to analyze DEGs were “resistant untreated × susceptible untreated” and “resistant 3 HAT × susceptible 3 HAT”. The annotation of the DEGs was based on the *E. crus-galli* *v.3* genome^7^ annotated by the International Weed Genomics Consortium (IWGC).^33^ Upregulated gene families known to metabolize herbicides in other studies, such as *CYP*, *GST*, *UGT*, *ABC*, and *AKR* were identified from the DEGs. The list of candidate genes was narrowed down by selecting genes from these families that were upregulated in the resistant biotype at both sampling time points (untreated and 3 HAT).

## 3 Results

### 3.1 Dose–Response Curves in Different Temperatures

The biotype SAOJER-R showed resistance to cyhalofop-butyl when exposed to 35/30 °C, surviving up to 570 g ha^–1^ (twice the herbicide label rate), but was susceptible at 25/20 °C, with 100% mortality at 142.5 g ha^–1^ (50% of the label rate) (Figure 1). The susceptible biotype VALE VERDE-S also increased its survival when exposed to higher temperatures, but at very low doses, surviving 8.9 g ha^–1^ at 25/20 °C, while up to 35.6 g ha^–1^ at 35/30 °C (Figure 1). When comparing the biotypes in each temperature, the resistance factor (RF) changed from 7–9 at 25/20 °C to 24–33 at 35/30 °C, depending on the experimental run (Table 2). This resistance phenotype response to temperature is an indication of non-target site metabolic resistance. The *ACCase* gene from resistant biotype SAOJER-R did not have any mutations at positions known to confer ACCase-inhibitor resistance (Figure S2).

**Figure 1:**
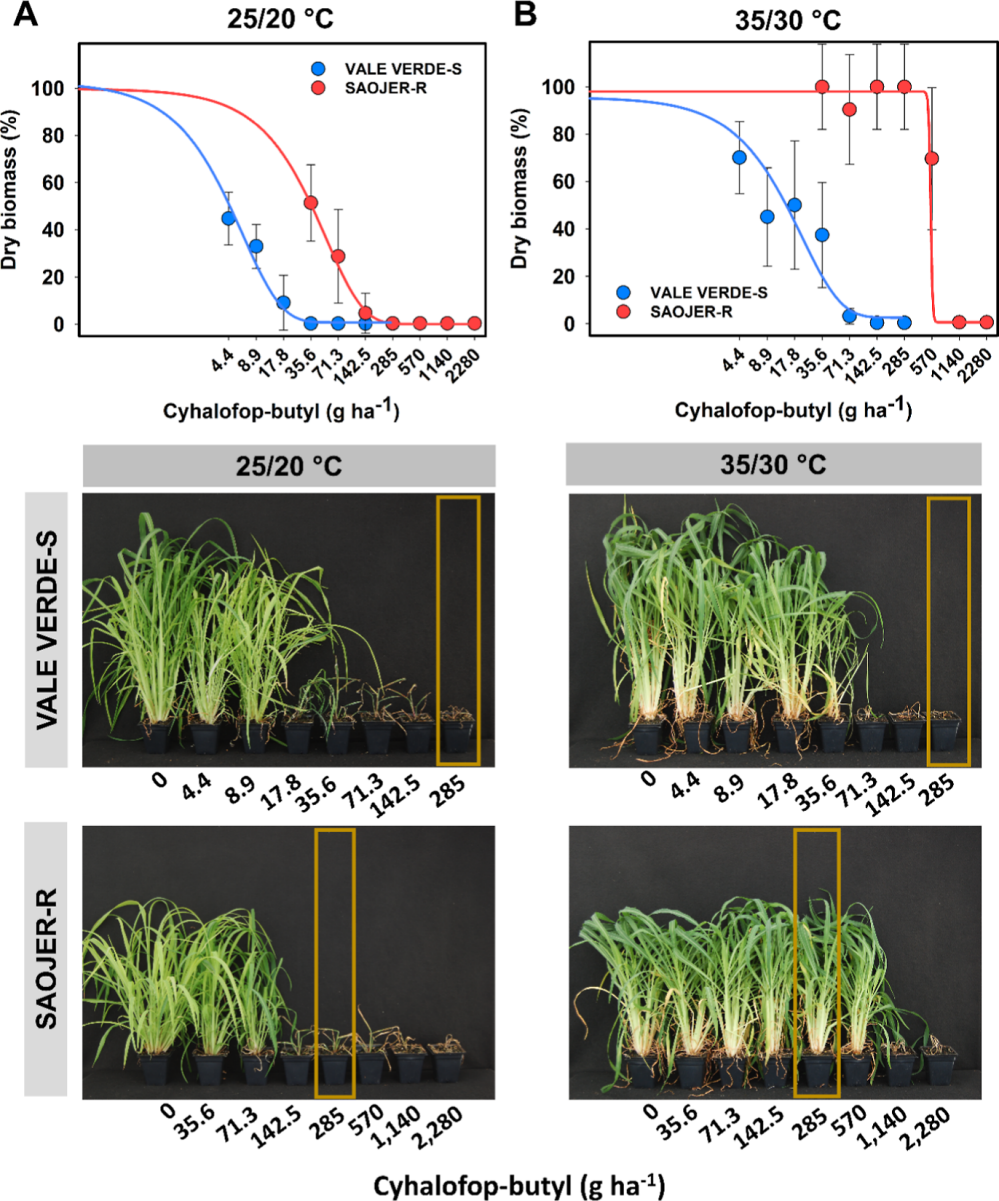
Cyhalofop-butyl dose–response curves of resistant (SAOJER-R) and susceptible (VALE VERDE-S) *E. crus-galli* biotypes performed at (A) 25/20 °C and (B) 35/30 °C, assessed at 28 DAT. Yellow boxes highlight the cyhalofop-butyl label rate (285 g ha^–1^). Vertical error bars indicate confidence interval (95% interval).

**Table 2:** Log-Logistic Cyhalofop-Butyl Dose–Response Curves Parameters of Two Experimental Runs with One *E. crus-galli* Resistant (SAOJER-R) and One Susceptible (VALE VERDE-S) Biotype Utilizing Dry Biomass Assessed at 28 DAT, and Performed at 25/20 °C or 35/30 °C

					*e*(GR_50_)		
biotype	temperature	*b*	*d*	*e*(GR_50_)	lower limit	upper limit	RF^a^
run 1							
VALE VERDE-S	25/20 °C	1.82*	6.54*	7.06*	5.31	8.81	
SAOJER-R	25/20 °C	1.18*	5.80*	51.92*	29.47	74.36	7.3
VALE VERDE-S	35/30 °C	7.31 ns	6.17*	21.34*	13.46	29.23	
SAOJER-R	35/30 °C	16.05 ns	5.22*	525.93*	–9.22	1061.09	24.6
run 2							
VALE VERDE-S	25/20 °C	1.39*	10.31*	4.21*	2.95	5.48	
SAOJER-R	25/20 °C	1.80*	8.13*	38.26*	27.61	48.92	9.1
VALE VERDE-S	35/30 °C	1.14*	7.39*	20.54 ns	–1.83	42.91	
SAOJER-R	35/30 °C	8.20 ns	5.09*	679.66*	280.66	1078.65	33.1

a RF: resistance factor; * Significant parameter according to the log-logistic three-parameter model fit (*p* ≤ 0.05); ns = non-significant parameter according to the three-parameter log-logistic model fit (*p* ≤ 0.05).

The duration of exposure to each temperature was reflected in the resistance phenotype. SAOJER-R plants grown at 35/30 °C were resistant only if kept for at least 48 HAT at high temperature. When plants grown at 35/30 °C were sprayed and moved to 25/20 °C growth chamber at 4, 8, or 24 HAT, they did not survive the herbicide cyhalofop-butyl application (Figure 2A,C). When the resistant plants were grown at 25/20 °C, the biomass decreased when increasing the exposure time to this temperature after cyhalofop-butyl application (Figure 2A,B). However, even if the plants were kept for 168 HAT at 25/20 °C before moving to 35/30 °C, it was not enough to reach a satisfactory control. The time of exposure to each temperature to reduce 50% of the biomass was estimated using the log-logistic model (Table 3 and Figure 2A). The biomass of SAOJER-R plants was reduced in 50% when exposed for 73 HAT at 25/20 °C before moving to 35/30 °C. Herbicide efficacy increases as the exposure time at 25/20 °C increases. On the other side, the exposure at 35/30 °C for 39 HAT before moving to 25/20 °C reduced 50% of the biomass, and the herbicide efficacy increased as the time of exposure to 35/30 °C decreased (Table 3).

**Figure 2:**
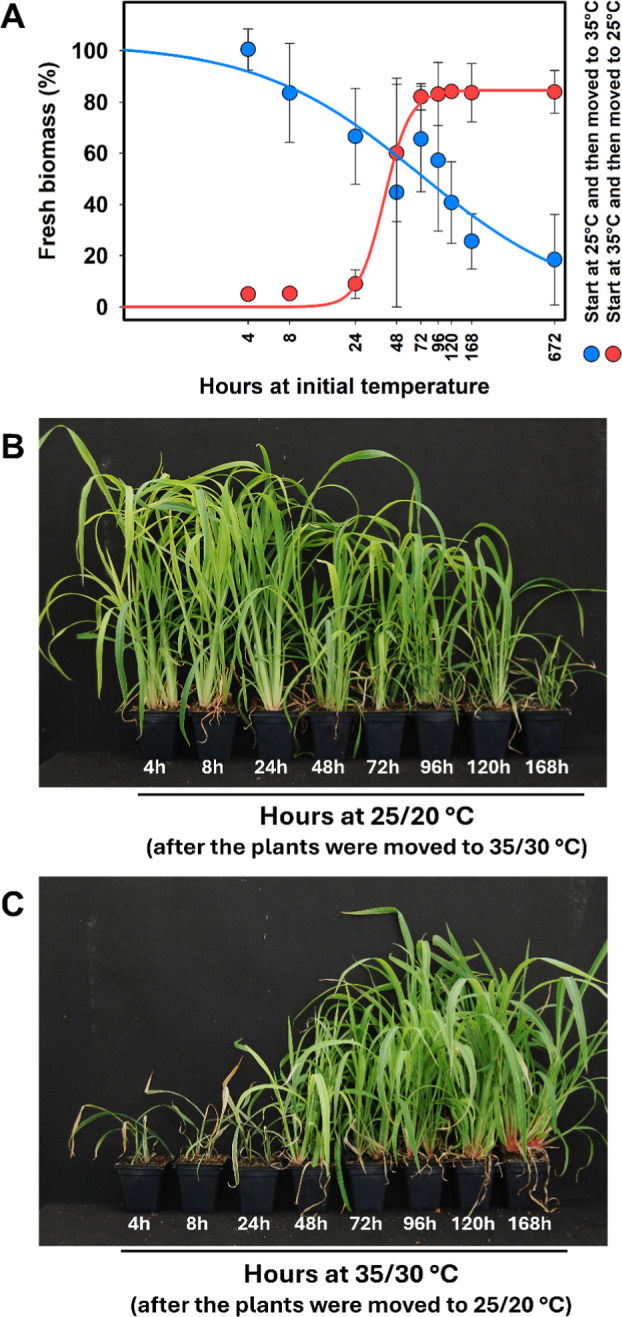
(A) Time–response curves of resistant biotype SAOJER-R exposed for different periods of time to 25/20 °C or 35/30 °C after 285 g ha^–1^ of cyhalofop-butyl application. Vertical error bars indicate confidence interval (95% interval). (B) SAOJER-R plants grown at 25/20 °C and moved to 35/30 °C at different HAT. (C) SAOJER-R plants grown at 35/30 °C and moved to 25/20 °C at different HAT.

**Table 3:** Log-Logistic Parameters of Time–Response Curve Utilizing Fresh Biomass of Resistant Biotype SAOJER-R Exposed during Different Hours after Cyhalofop-Butyl Application to 25/20 °C or 35/30 °C

				*e*(time_50_)	
	*b*	*d*	*e*(time_50_)	lower limit	upper limit
start at 35/30 °C and then moved to 25/20 °C	–4.57^ns^	84.63*	39.02*	23.91	54.12
start at 25/20 °C and then moved to 35/30 °C	0.72*	102.93*	73.35*	–1.72	148.43

*Significant parameter according to the log-logistic three-parameter model fit (*p* ≤ 0.05); ns = non-significant parameter according to the three-parameter log-logistic model fit (*p* ≤ 0.05); time_50_ = duration in hours at a specific temperature to reduce 50% of the fresh biomass.

### 3.2 Quantification of Cyhalofop-Butyl and Cyhalofop Acid in Plants

The quantification of cyhalofop-butyl (parent compound) and cyhalofop acid (active compound) showed faster metabolism in the resistant biotype SAOJER-R compared to the susceptible at 35/30 °C (Figure 3A). The amount of cyhalofop-butyl in resistant and susceptible biotypes did not differ at 8 and 24 HAT at 25/20 °C, but at 72 HAT, there was 50% less cyhalofop-butyl in the resistant than in the susceptible. At 35/30 °C, the amount of cyhalofop-butyl quantified in the resistant was 57% and 75% less than in the susceptible at 8 and 24 HAT, respectively. When comparing the temperatures, the amount of cyhalofop-butyl in the resistant biotype was 58%, 92%, 82%, 85%, and 100% less under 35/30 °C than 25/20 °C at 8, 24, 72, 120, and 168 HAT, respectively. For the susceptible biotype VALE VERDE-S, a smaller amount of cyhalofop-butyl was found under 35/30 °C than at 25/20 °C (Figure 3A). For the active compound cyhalofop acid, the amount quantified in SAOJER-R at 25/20 °C was 53%, 42%, and 46% less than in the VALE VERDE-S at 8, 24, and 72 HAT, respectively. At 35/30 °C, cyhalofop acid was 49%, 71%, 50%, 67%, and 95% less in the resistant compared to the susceptible at 8, 24, 72, 120, and 168 HAT, respectively (Figure 3B). Comparing the temperatures, the resistant biotype had 70% and 43% less cyhalofop acid at 24 and 72 HAT, respectively, under 35/30 °C than at 25/20 °C. The susceptible biotype also had smaller amount of cyhalofop acid at 8, 24, and 72 HAT under 35/30 °C than at 25/20 °C (Figure 3B). These results corroborate the different responses verified in the dose–response curves performed at two different temperatures.

**Figure 3:**
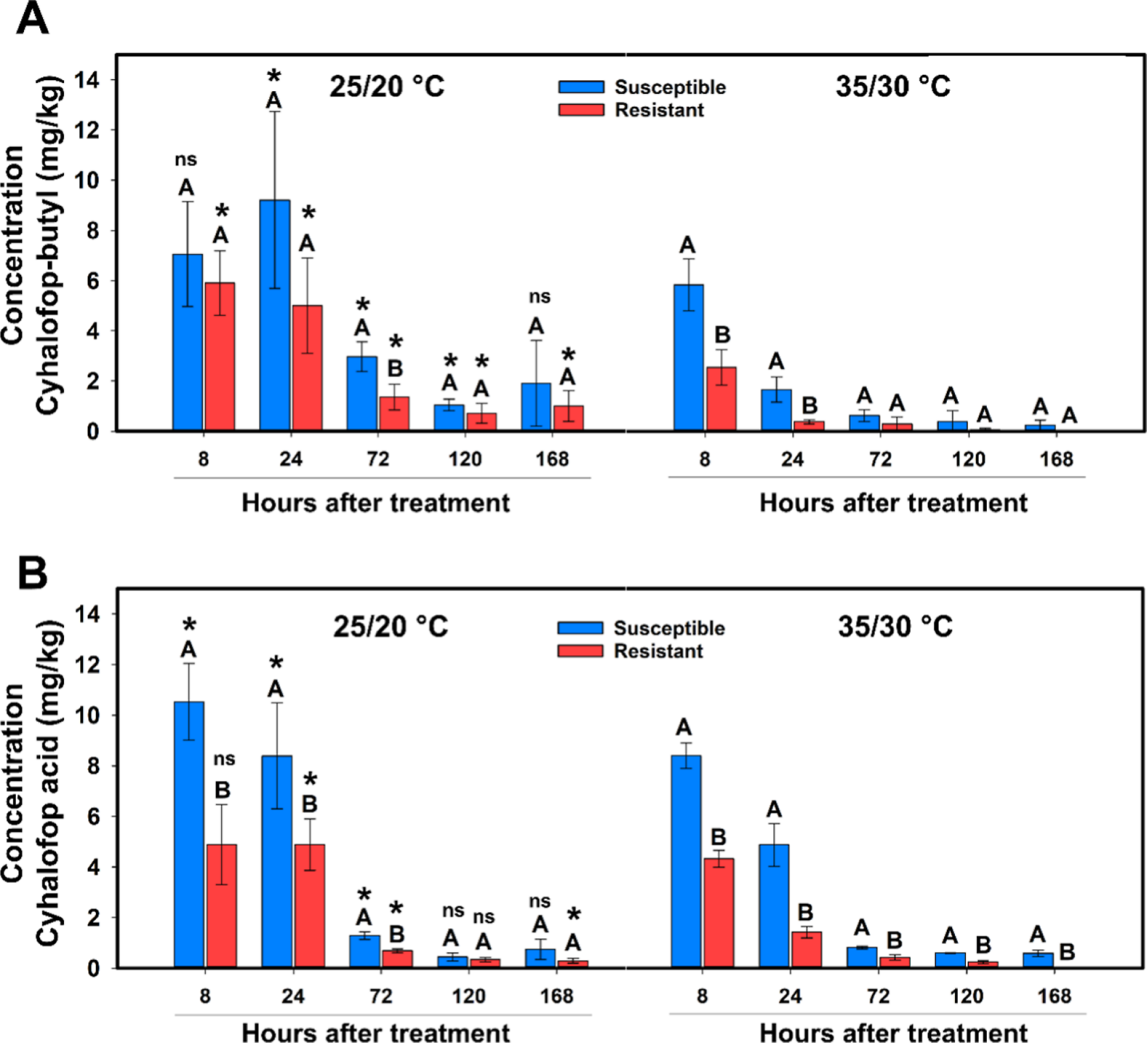
LC–MS/MS quantification of (A) cyhalofop-butyl and (B) cyhalofop acid in resistant (SAOJER-R) and susceptible (VALE VERDE-S) *E. crus-galli* biotypes grown at 25/20 °C or 35/30 °C and sampled at different time points after cyhalofop-butyl herbicide application at 285 g ha^–1^. Vertical error bars indicate confidence interval (95% interval). Capital letters compare the two biotypes at the same sampling time point and at the same temperature. * or ns compares the same biotype at the same time point but at different temperatures according to the *t*-test (α = 0.05).

The potential involvement of CYP450 in cyhalofop metabolism was evaluated by treating the plants with malathion, a CYP450 inhibitor, at both temperatures. The amount of cyhalofop-butyl was about 10 and 18 times higher at 72 and 120 HAT, respectively, with malathion application in the resistant and susceptible biotypes under 25/20 °C. The cyhalofop acid amount increased from 7 to 14× higher in both resistant and susceptible at 72 and 120 HAT biotypes when malathion was sprayed under 25/20 °C (Figure 4A,C). Malathion reduced the plant’s ability to metabolize the herbicide, as measured by increased herbicide amount in plants at the sampling time points, suggesting that a CYP450 is involved in the degradation of cyhalofop. No differences were observed at 35/30 °C for the resistant biotype (Figure 4B,D). This may indicate that the malathion dose was insufficient to inhibit all CYP450 activity in the experiment at 35/30 °C.

**Figure 4:**
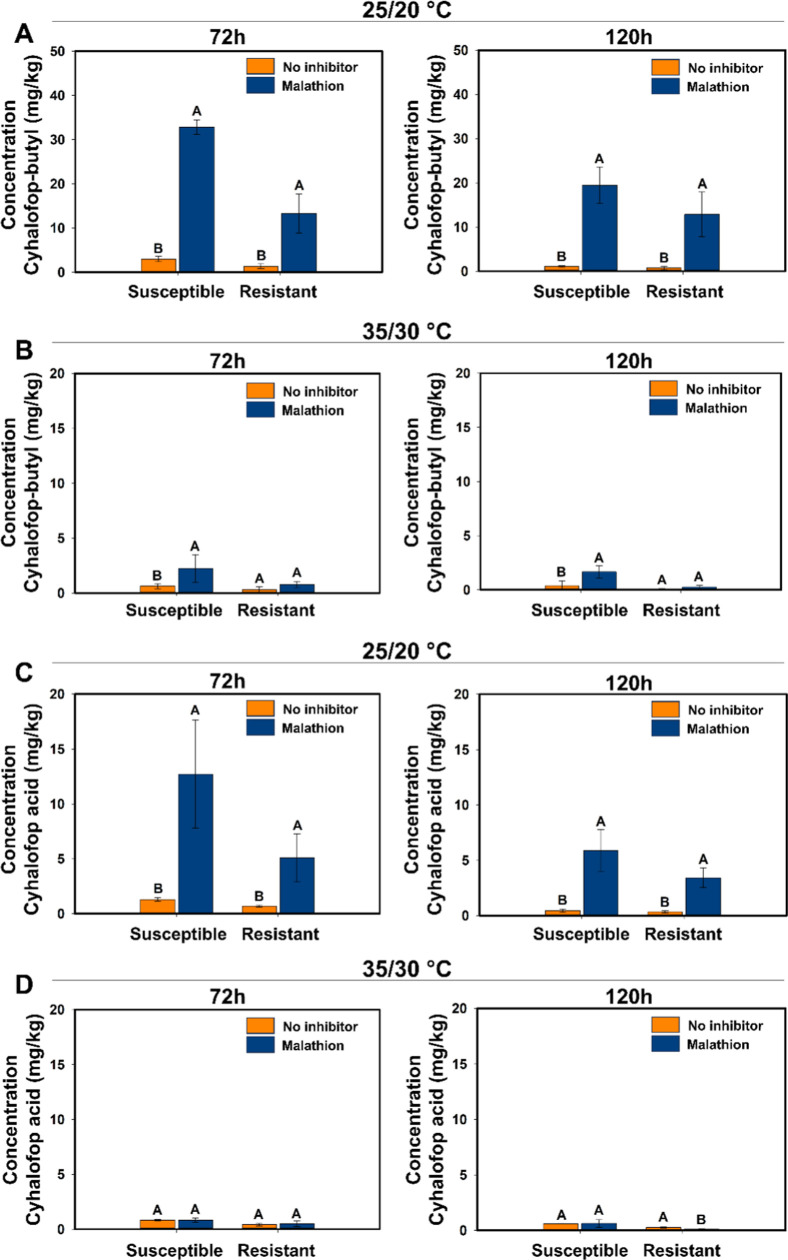
LC–MS/MS quantification of cyhalofop-butyl (A,B) and cyhalofop acid (C,D) with and without malathion (CYP450 inhibitor) application grown at (A,C) 25/20 °C or (B,D) 35/30 °C in resistant (SAOJER-R) and susceptible (VALE VERDE-S) *E. crus-galli* biotypes sampled at 72 and 120 h after cyhalofop-butyl herbicide application at 285 g ha^–1^. Vertical error bars indicate confidence interval (95% interval). Capital letters compare the amount of compound with and without malathion in the same biotype.

### 3.3 Differentially Expressed Genes in RNA-seq Analysis

The total RNA was extracted and sequenced at two sampling time points to evaluate the DEGs in the resistant biotype SAOJER-R compared to the susceptible VALE VERDE-S. Considering a cutoff at least twice upregulation or downregulation, the SAOJER-R had 1123 upregulated and 3059 downregulated at untreated contrast, and 7833 upregulated and 8659 downregulated at 3 HAT (Figure 5A). When analyzing the contrasts, 624 genes (7.5%) were upregulated and 811 (7.4%) were downregulated in both sampling times (untreated and at 3 HAT) (Figure 5B). Gene families already known to metabolize several herbicide chemical classes (i.e., *CYP*, *GST*, *UGT*, *ABC*, and *AKR*) were searched among the upregulated genes shared by the RNA-seq contrasts. The contrast “resistant untreated × susceptible untreated” showed eight *CYP*, seven *GST*, 13 *UGT*, four *ABC*, and two *AKR* genes upregulated in the resistant biotype, while the contrast “resistant 3 HAT × susceptible 3 HAT” showed 111 *CYP*, 71 *GST*, 53 *UGT*, 63 *ABC*, and 18 *AKR* genes. This indicates a quick upregulation of many metabolic genes in response to cyhalofop-butyl application. However, only seven *CYP*, seven *GST*, five *UGT*, two *ABC*, and two *AKR* genes were upregulated in the resistant biotype compared to the susceptible in both RNA-seq contrasts (Figure 6 and Table 4). The LFC of *CYP* genes in the resistant biotype ranged from 2.1 to 4.3 at 3 HAT. The *GST* and *UGT* gene families showed the highest upregulated genes in SAOJER-R treated with cyhalofop-butyl compared to the susceptible VALE VERDE-S treated. The *GST* coding genes BH02.4558, BH01.4569, and BH01.2429 showed LFC of 15.1, 7.9, and 6.8, respectively, while the *UGT* gene BH03.3274 showed LFC of 10.1 (Figure 6 and Table 4). The gene *ACCase* was not differentially expressed between the biotypes, which discards the occurrence of herbicide target increased expression or copy number variation.

**Figure 5:**
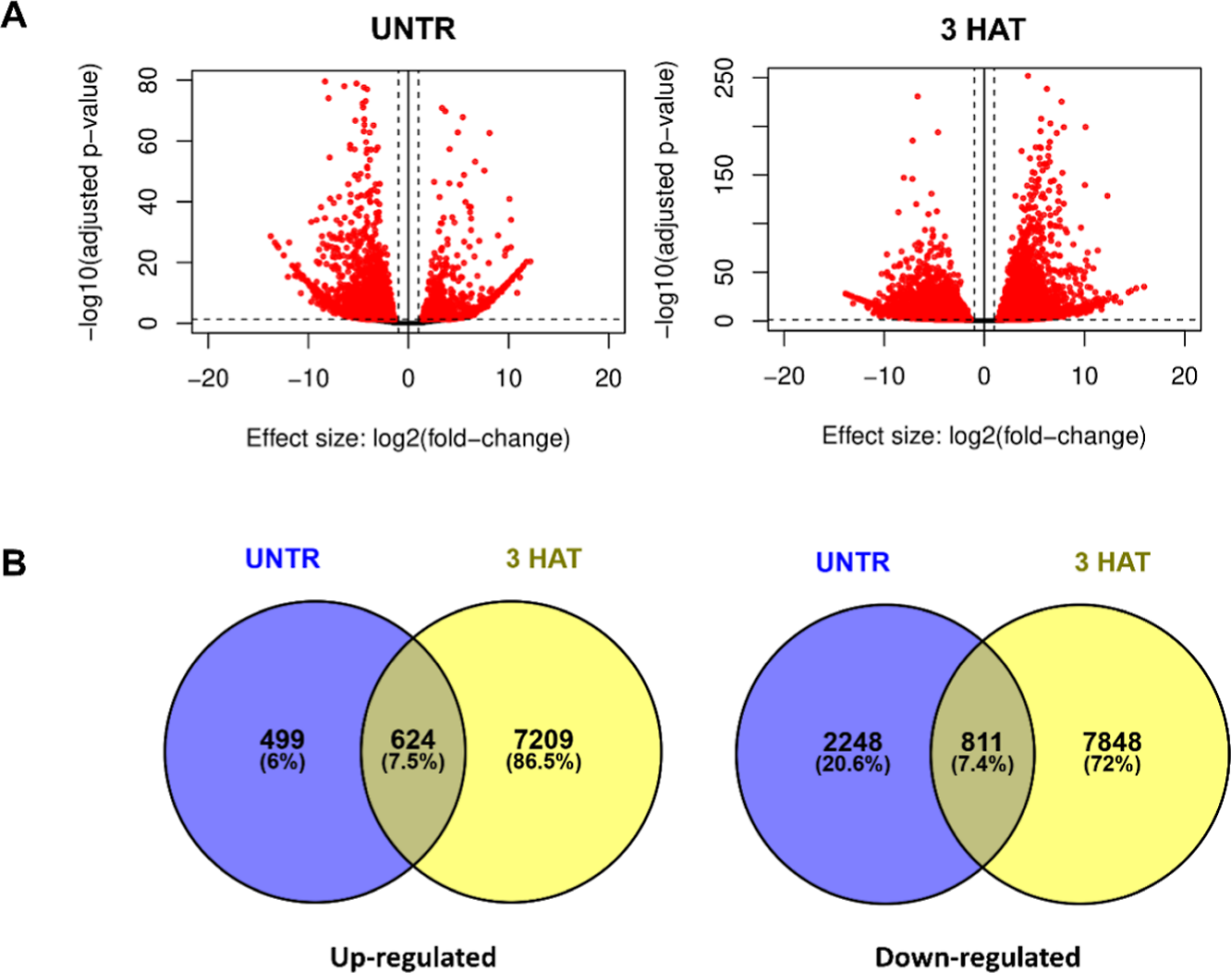
(A) Volcano-plot of RNA-seq contrasts (“resistant untreated × susceptible untreated” and “resistant 3 HAT × susceptible 3 HAT”) showing the genes upregulated and downregulated in the resistant biotype compared to the susceptible. (B) Venn diagram of genes upregulated and downregulated shared among the contrasts.

**Figure 6:**
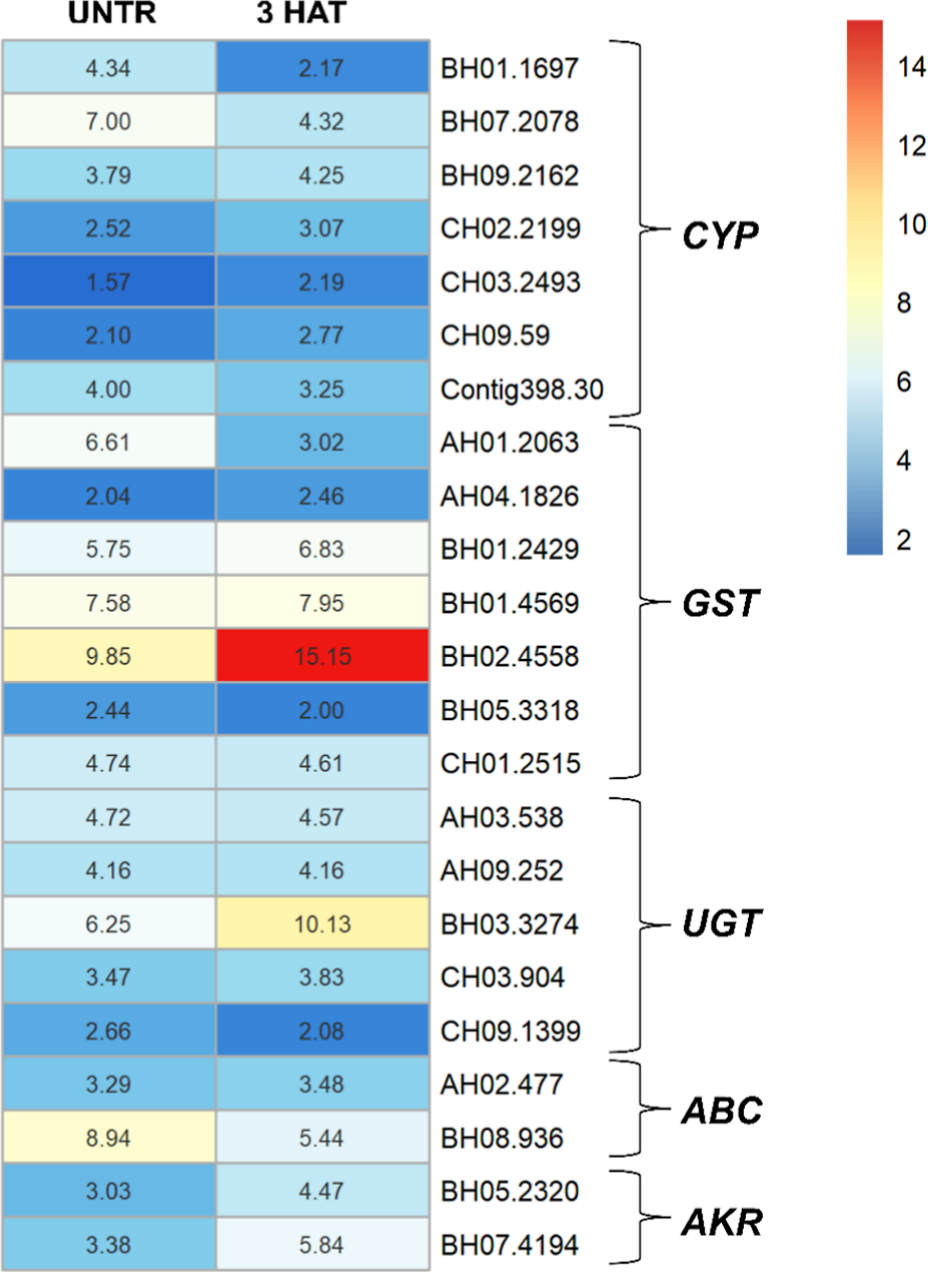
Heatmap of genes from families known to metabolize herbicides found upregulated in the resistant biotype SAOJER-R in both RNA-seq contrasts, “resistant untreated × susceptible untreated” and “resistant 3 HAT × susceptible 3 HAT”.

**Table 4:** Functional Annotation, Log2 Fold Change (LFC), and Adjusted *p*-value of the Genes from Families Known to Metabolize Herbicides Found Upregulated in the Resistant Biotype Compared to the Susceptible (Grown at 35/30 °C) and Shared by Both RNA-seq Contrasts (Untreated (UNTR) and at 3 HAT)

		UNTR		3 HAT		
family	genes	LFC	adj *p*-value	LFC	adj *p*-value	functional annotation
*CYP*	BH01.1697	4.34	1.2 × 10^–4^	2.17	4.7 × 10^–2^	*tyrosine N-monooxygenase-like*
	BH07.2078	7.00	1.9 × 10^–5^	4.32	4.0 × 10^–3^	*CYP86B1-like*
	BH09.2162	3.79	1.7 × 10^–7^	4.25	1.4 × 10^–11^	*CYP704C1*
	CH02.2199	2.52	8.7 × 10^–5^	3.07	2.4 × 10^–4^	*CYP87A3*
	CH03.2493	1.57	4.1 × 10^–2^	2.20	3.0 × 10^–7^	*abscisic acid 8′-hydroxylase 3 isoform X2* (*CYP707A3*)
	CH09.59	2.10	3.9 × 10^–2^	2.77	1.3 × 10^–3^	*CYP93G1*-like
	Contig398.30	4.00	1.2 × 10^–3^	3.25	3.0 × 10^–2^	*indole-2-monooxygenase*
*GST*	AH01.2063	6.61	3.7 × 10^–4^	3.02	4.1 × 10^–2^	probable *glutathione S-transferase BZ2*
	AH04.1826	2.05	1.4 × 10^–3^	2.46	1.7 × 10^–6^	*glutathione S-transferase T1*
	BH01.2429	5.75	9.4 × 10^–4^	6.83	4.6 × 10^–5^	probable *glutathione S-transferase BZ2*
	BH01.4569	7.58	2.5 × 10^–7^	7.95	1.5 × 10^–8^	*glutathione S-transferase T3-like isoform X2*
	BH02.4558	9.85	2.8 × 10^–25^	15.16	3.9 × 10^–34^	probable *glutathione S-transferase*
	BH05.3318	2.44	7.8 × 10^–9^	2.00	2.3 × 10^–5^	*glutathione S-transferase T3*-like
	CH01.2515	4.74	3.9 × 10^–3^	4.61	1.2 × 10^–3^	probable *glutathione S-transferase BZ2*
*UGT*	AH03.538	4.72	8.1 × 10^–9^	4.57	9.7 × 10^–6^	*UDP-glycosyltransferase 91C1*
	AH09.252	4.16	3.0 × 10^–7^	4.16	5.9 × 10^–27^	*crocetin glucosyltransferase 2*
	BH03.3274	6.25	3.6 × 10^–35^	10.13	4.2 × 10^–18^	*UDP-glycosyltransferase 91B1*-like
	CH03.904	3.47	1.8 × 10^–11^	3.83	6.1 × 10^–13^	*DIMBOA UDP-glucosyltransferase BX9*-like
	CH09.1399	2.66	1.4 × 10^–13^	2.08	1.8 × 10^–6^	*7-deoxyloganetin glucosyltransferase*-like
*ABC*	AH02.477	3.29	5.9 × 10^–20^	3.48	1.4 × 10^–28^	*ABC transporter C family member 3*
	BH08.936	8.94	1.5 × 10^–29^	5.44	3.5 × 10^–35^	*ABC transporter G family member 17-like* (Low quality protein)
*AKR*	BH05.2320	3.03	9.1 × 10^–9^	4.47	3.1 × 10^–18^	*aldo-keto reductase family 4 member C10*-like
	BH07.4194	3.38	3.7 × 10^–16^	5.84	4.6 × 10^–44^	*NADPH-dependent aldo-keto reductase chloroplastic*-like

## 4 Discussion

The biotype SAOJER-R is resistant to cyhalofop-butyl due to enhanced metabolic activity in a temperature-dependent manner, with a susceptible response at 25/20 °C and a resistant response at 35/30 °C. High temperatures make weeds less sensitive to herbicides, likely in part due to increased overall plant enzymatic activity, including the enzymes involved in herbicide metabolism.^34^ The susceptible biotype VALE VERDE-S also had increased GR_50_ at 35/30 °C compared to 25/20 °C. The effect of high temperature increasing the RF of resistant populations has been shown in other metabolic-resistant weed species, such as in *E. colona* resistant to glyphosate, where no target site resistance mechanisms were identified, and the resistant phenotype was verified at 30 °C but not at 25 °C;^35^ *Amaranthus palmeri* resistant to 2,4-D had an RF increased 10 times when the temperature increased from 24/14 °C to 34/24 °C;^23^ *Conyza bonariensis* and *C. canadensis* resistant to glyphosate had the resistance reversed at low temperatures (12 °C).^24,25^ However, the *Hordeum leporinum* resistant to paraquat shows the opposite behavior, resistant at cool temperatures and susceptible at high temperatures.^36^ Therefore, the environmental conditions, especially temperature, become an important factor for field resistance diagnostics, greenhouse studies, or herbicide resistance modeling, which has not been verified yet in several recently developed models.^37–39^ The complex interaction of the resistance mechanism with temperature changes does not reflect in a straightforward practical recommendation for resistance management. Resistant plants must be maintained for several days (>7 days) at 25/20 °C or cooler to achieve effective control with cyhalofop-butyl application. Considering that 25/20 °C is still warm and a common average temperature during early rice season, it could be beneficial for resistance management if it lasts for several days. However, the occurrence of periods of high temperature, even if starting at 5 DAT for example, will allow plants to survive. The direct temperature effect on resistant phenotype is reflected in variability of cyhalofop-butyl resistance phenotype in the field. The dose of cyhalofop-butyl utilized by farmers to efficiently control *Echinochloa* spp. in the field has been increasing in the last 12 years, doubling the label rate, which correlates with the average global temperature increase. The 10 warmest years in the historical record have occurred from 2014 to 2023.^40^ The positive correlation between resistance level and temperature is an indication of climate change consequences in future weed management.^41^ Weeds may become more resistant to herbicides than they currently are, and susceptible plants may also require higher doses to be killed.

The exposure of 35/30 °C for 24 h was not enough time for the resistant biotype SAOJER-R to metabolize cyhalofop-butyl efficiently to avoid being killed. SAOJER-R needed at least 48 h at 35/30 °C after application to survive. However, the first 48 HAT were not critical when at 25/20 °C condition, because even after 7 days continuously at 25/20 °C and then moved to 35/30 °C for 21 additional days, SAOJER-R survived the cyhalofop-butyl application, indicating the importance of a late metabolism in metabolic resistance. We conclude that there was not enough herbicide reaching the target site to kill the plant during the first 7 days at 25/20 °C in the resistant biotype, but there is a negative correlation between time at 25/20 °C and fresh biomass. The temperature may affect foliar herbicide absorption^42^ and is known to change the cell membrane properties, including permeability.^43^ The temperature of 25/20 °C may decrease the herbicide absorption and/or decrease the membrane permeability compared to 35/30 °C, slowing down the cyhalofop-butyl entrance in the cell, which helps to not overload the detoxification system of the resistant biotype for the first 7 days. We suggest a model in which if the temperature does not increase after a certain period of time, the herbicide will keep passing the membranes and its accumulation in the cell will overload the metabolic detoxification system, and some herbicide amount will reach the ACCase target site. In *Amaranthus tuberculatus*, 7% of the initially applied 2,4-D was present in resistant plants at 11 days after application, while susceptible plants still had 25%.^44^ This information sheds light on the different time periods that plants take to detoxify herbicides when interacting with different environmental conditions, and the importance of the late herbicide detoxification, which is often neglected.

The application of CYP450 inhibitor in SAOJER-R plants grown at 25/20 °C increased the amount of cyhalofop-butyl and cyhalofop acid quantified in both resistant and susceptible biotypes, indicating an important role of these enzymes in cyhalofop-butyl detoxification. However, the effect was different in plants grown at 35/30 °C, which could be explained by an insufficient inhibitor dose in the high enzymatic activity condition. This hypothesis is supported by another study, which verified that malathion increased the susceptibility of a 2,4-D-resistant *A. palmeri* population when high temperatures were combined with high rates of malathion. In that study, malathion was applied twice: 1500 g ai ha^–1^ 30 min before the herbicide, followed by 50 mL pot^–1^ of 5 mM malathion 48 h after herbicide application.^23^ Nevertheless, we cannot rule out factors beyond, or in addition to, the specific CYP450 pathway.

The biotype SAOJER-R is metabolically resistant to cyhalofop-butyl, as proven by the absence of mutation in *ACCase* genes, faster metabolism of cyhalofop compounds, resistance affected by temperature, and upregulation of herbicide detoxification gene families. Seven *CYP*s, seven *GST*s, five *UGT*s, two *ABC*s, and two *AKR*s were upregulated in SAOJER-R compared to the susceptible VALE VERDE-S in both sampling time points, untreated, and at 3 HAT. However, many more genes from these metabolic families were upregulated in SAOJER-R specifically at 3 HAT compared with the untreated contrast, indicating that multiple metabolic families responded to cyhalofop-butyl application and may play a role in its metabolism in SAOJER-R. Among the genes upregulated in both RNA-seq contrasts, three *GST* genes and one *UGT* gene showed the highest upregulation. Several genes from *CYP*, *GST*, and *UGT* families have already been reported to participate in ACCase inhibitor degradation in other studies. The genes *CYP81A68* ^17^ and *GSTU23* ^12^ were validated in metabolizing cyhalofop-butyl in *E. crus-galli*, while *CYP709C9*,^19^ *CYP81A12*, and *CYP81A21* ^20^ metabolize other ACCase herbicides in *Echinochloa* sp., such as diclofop-methyl. Of seven *CYP* genes upregulated in SAOJER-R, three have already been associated with herbicide resistance in other studies. The *CYP87A3* was reported as a candidate gene for fenoxaprop-*p*-ethyl (ACCase inhibitor) resistance in *Beckmannia syzigachne*;^45^ *CYP86B1* was the main candidate gene for ALS inhibitor mesosulfuron-methyl in *B. syzigachne* ^46^ and tribenuron-methyl resistance in *Myosoton aquaticum*;^47^ *CYP704C1* is able to metabolize fenoxaprop-*p*-ethyl, mesosulfuron-methyl, and isoproturon (Photosystem II inhibitor) also in *B. syzigachne*.^48^ The identification of herbicide resistance due to enhanced metabolism by CYP450 in *E. crus-galli* has been shown to increase the susceptibility to other herbicides, such as clomazone, which is important for metabolic resistance management.^49^ Some of the upregulated genes from other families were also already associated with herbicide resistance. The gene *glutathione S-transferase T3*-like is involved in fenoxaprop-*p*-ethyl metabolism in *B. syzigachne*;^50^ *UDP-glycosyltransferase 91C1* catalyzes the glucosylation of sulcotrione (HPPD inhibitor) in *Arabidopsis*;^51^ *aldo-keto reductase family 4 member C10-*like is involved in glyphosate resistance in *E. colona* ^10^ and *Lolium rigidum*;^52^ and *ABC transporter G family member 17*-like was also upregulated in another *E. crus-galli* resistant to cyhalofop-butyl.^18^

The other candidate genes found upregulated have not been associated with herbicide resistance in any study yet, but they have other metabolic roles. *CYP93G1* is involved in flavonoid metabolism, catalyzing the direct conversion of flavanones to flavones.^53^ *Abscisic acid 8′-hydroxylase 3* (also known as *CYP707A3*) catalyzes the oxidative degradation of abscisic acid and is a crucial enzyme controlling the rate of degradation of this plant hormone.^54,55^ *Glutathione S-transferase BZ2* has a role in anthocyanin biosynthesis, conjugating it with glutathione.^56^ Glucosylation by an *UDP-glucosyltransferase BX9* is essential for the reduction of plant autotoxicity of the benzoxazinoids.^57^ The commitment of these genes to a primary function in plant metabolism does not prevent them from being involved also in xenobiotic degradation, such as herbicides.

The biotype SAOJER-R is resistant to cyhalofop-butyl because of the interaction of metabolic gene families upregulation with high temperature 35/30 °C condition. Faster cyhalofop-butyl and cyhalofop acid metabolism were verified in the resistant biotype when compared to the susceptible biotype. *CYP*s are candidate genes because the application of CYP450 inhibitor increased the amount of herbicide compounds in planta. However, genes from *GST* and *UGT* showed higher expression. The susceptibility of SAOJER-R to cyhalofop-butyl at label rate sprayed at 25/20 °C raises the question about the standardization of conditions to evaluate metabolic herbicide resistance. The 25/20 °C temperature program is commonly utilized when screening resistance in greenhouse or growth chambers, and depending on whether this temperature is similar or different from the temperature conditions experienced by the biotype in the field, false negatives (concluding susceptibility when resistance is present) may be possible. A relatively short period of 48 h at 35/30 °C was sufficient to metabolize the herbicide and prevent its activity in killing the resistant biotype, but up to 7 days at 25/20 °C was not enough to restore the susceptibility, which requires longer periods at 25/20 °C. We emphasize that 25°/20 °C is not considered low temperature. This is the first study reporting susceptibility at warm and resistance at high temperature, which may be considered to manage cyhalofop-butyl resistance in the field, especially for early rice season planting.

## Supplementary Material



## Data Availability

RNA-seq short-reads data are available under BioProject PRJNA1366807 in GenBank (accessions SRR36107918, SRR36107922–SRR36107932). All other data are provided in the manuscript or Supporting Information. Additional data sets will be made available upon reasonable request.

## Notes

Partial *ACCase* genes from all three *E. crus-galli* subgenomes; alignment of the partial *ACCase* genes showing primers targeting (yellow: ACCase1F–ACCase1R; light blue: ACCase3F–ACCase3R; green: ACCase7F–ACCase7R); red indicating the position where mutations conferring herbicide resistance are found; * indicating the same nucleotide in all three subgenomes; absence of * indicating there is at least one different nucleotide between them; sequencing of *ACCase* genes in cyhalofop-butyl susceptible and resistant biotypes (highlighted positions known to confer herbicide resistance when amino acid substitutions occur); and LC–MS/MS settings utilized to detect cyhalofop-butyl and cyhalofop acid (PDF)

## Abbreviations

acetolactate synthase: (ALS)
acetyl-CoA carboxylase: (ACCase)
cytochrome P450 monooxygenases: (CYP and CYP450)
ATP-binding cassette: (ABC)
aldo-keto reductases: (AKR)
UDP-glycosyltransferase: (UGT)
glutathione-*S*-transferase: (GST)
differentially expressed genes: (DEGs)
resistance factor: (RF)
dose that reduces 50% of the dry biomass: (GR_50_)
analysis of variation: (ANOVA)
days after treatment: (DAT)
hours after treatment: (HAT)
methylated seed oils: (MSO)
duration in hours at a specific temperature to reduce 50% of the fresh biomass: (time_50_)
