# Supplementary material for: High Temperatures Are Required for Expression of Metabolic Resistance to Cyhalofop-Butyl in a Biotype of Echinochloa crus-galli
Source: J Agric Food Chem. 2026 Apr 3;74(14):11461–72. doi: 10.1021/acs.jafc.5c16988 (PMC13088228; doi:10.1021/acs.jafc.5c16988)
Supplement: Supplementary file 1 [file jf5c16988_si_001.pdf]

SUPPORTING INFORMATION

High temperatures are required for expression of metabolic resistance to cyhalofop-butyl in a biotype of *Echinochloa crus-galli*

Luan Cutti[a], André Lucas Simões Araujo[b], Guilherme Menegol Turra[c], Carlos Alberto Gonsiorkiewicz Rigon[d], Paula Sinigaglia Angonese[e], Mateus Gallon[f], Franck Dayan[g], Todd Gaines[h], Aldo Merotto[i*].

[a] Department of Crop Science, Federal University of Rio Grande do Sul, Porto Alegre, RS, Brazil, 91540-000.

[b] Department of Agricultural Biology, Colorado State University, Fort Collins, CO, USA, 80523.

[c] Department of Crop Science, Federal University of Rio Grande do Sul, Porto Alegre, RS, Brazil, 91540-000.

[d] Department of Agricultural Biology, Colorado State University, Fort Collins, CO, USA, 80523.

[e] Department of Crop Science, Federal University of Rio Grande do Sul, Porto Alegre, RS, Brazil, 91540-000.

[f] Department of Crop Science, Federal University of Rio Grande do Sul, Porto Alegre, RS, Brazil, 91540-000.

[g] Department of Agricultural Biology, Colorado State University, Fort Collins, CO, USA, 80523.

[h] Department of Agricultural Biology, Colorado State University, Fort Collins, CO, USA, 80523.

[i] Department of Crop Science, Federal University of Rio Grande do Sul, Porto Alegre, RS, Brazil, 91540-000.

*Corresponding author: aldo.merotto@ufrgs.br

Table of contents

Page S-3 - S-5

Suppl. Fig. S1. Partial *ACCase* genes from all three *E. crus-galli* subgenomes. Alignment of the partial *ACCase* genes showing primers targeting (Yellow: ACCase1F– ACCase1R; Light blue: ACCase3F – ACCase3R; Green: ACCase7F – ACCase7R). Red indicates the position where mutations conferring herbicide resistance are found. Asterisk (*) indicates the same nucleotide in all three subgenomes. Absence of * indicates there is at least one different nucleotide between them.

Page S-6

Suppl. Fig. S2. Sequencing of *ACCase* genes in cyhalofop-butyl susceptible and resistant biotypes. The highlighted positions are known to confer herbicide resistance when amino acid substitutions occur.

Page S-7

Suppl. Table S1. LC-MS/MS settings utilized to detect cyhalofop-butyl and cyhalofop acid

Supplementary Figures

```
BH05.2894      AATGAAGAAGACTATGCGCGTATTAGCTCTTCTGTTATAGCACACAAGCTGCAGCTGGAT  5259
CH05.2958      AATGAAGAAGACTATGCGCGTATTAGCTCTTCTGTTATAGCACACAAGCTGCAGCTGGAT  5259
AH09.362       ACTGAAGAAGACTATGATCGTATTAGATCTTCTGTTATAGCACACAAGCTGCAGCTGGAT  5259
BH09.424       ACTGCAGAAGACTATGATCGTATTAGCTCTTCTGTTATAGCACACAAGGTGCAGCTGGAT  6825
CH09.447       ACTGAAGAAGACTATGATCGTATTAGCTCTTCTGTTATAGCACACAAGCTGCAGCTGGAT  5256
               * ** ***********  ******** ********************* ***********

BH05.2894      AGTGGGGAAACTAGGTGGATCATTGACTCCGTTGTCGGCAAGGAGGATGGGCTTGGTGTT  5319
CH05.2958      AGTGGGGAAACTAGGTGGATCATTGACTCCGTTGTCGGCAAGGAGGATGGGCTTGGTGTT  5319
AH09.362       AGTGGTGAAATTAGGTGGATTATTGACTCTGTTGTGGGCAAGGAGGATGGTCTTGGTGTT  5319
BH09.424       AGTGGTGAAGTTAGGTGGATTATTGACTCTGTTGTGGGCAAGGAGGATGGTCTTGGTGTT  6885
CH09.447       AGTGGTGAAGTTAGGTGGATTATTGACTCTGTTGTGGGCAAGGAGGATGGTCTTGGTGTT  5316
               ***** ***  ********* ******** ***** ************** *********

BH05.2894      GAGAATATACATGGAAGTGCTGCTATTGCCAGAGCTTATTCTAGGGCATATGAGGAGACA  5379
CH05.2958      GAGAATATACATGGAAGTGCTGCTATTGCCAGAGCTTATTCTAGGGCATATGAGGAGACA  5379
AH09.362       GAGAATATACATGGAAGTGCTGCTATTGCCAGTGCTTATTCTAGGGCATATAAGGAGACA  5379
BH09.424       GAGAATATACATGGAAGTGCTGCTATTGCCAGTGCTTATTCTAGGGCATATGAGGAGACA  6945
CH09.447       GAGAATATACATGGAAGTGCTGCTATTGCCAGTGCTTATTCTAGGGCATATGAGGAGACA  5376
               ******************************** ****************** ********

BH05.2894      TTTACACTTACATTTGTGACTGGGCGGACTGTTGGAATAGGAGCTTATCTTGCTCGGCTT  5439
CH05.2958      TTTACACTTACATTTGTGACTGGGCGGACTGTTGGAATAGGAGCTTATCTTGCTCGGCTT  5439
AH09.362       TTTACACTTACATTCGTGACTGGGCGGACTGTAGGAATAGGAGCTTATCTTGCTCGGCTT  5439
BH09.424       TTTACACTTACATTCGTGACTGGACGGACTGTAGGAATAGGAGCTTATCTTGCTCGGCTT  7005
CH09.447       TTTACACTTACATTCGTGACTGGGCGGACTGTAGGAATAGGAGCTTATCTTGCTCGGCTT  5436
               ************** ******** ******** ***************************

BH05.2894      GGTATACGGTGCATACAACGTCTTGACCAGCCTATTATTTTAACTGGTTTTTCAGCCCTG  5499
CH05.2958      GGTATACGGTGCATACAACGTCTTGACCAGCCTATTATTTTAACTGGGTTTTCAGCCCTG  5499
AH09.362       GGTATACGGTGCATACAGCGTCTAGACCAGCCTATTATTTTAACTGGGTTTTCTGCCCTG  5499
BH09.424       GGTATACGGTGCATACAGCGTCTAGACCAGCCTATTATTTTAACTGGGTTTTCTGCCCTG  7065
CH09.447       GGTATACGGTGCATACAGCGTCTAGACCAGCCTATTATTTTAACCGGGTTTTCTGCCCTG  5496
               ***************** ***** ******************** ** ***** ******

BH05.2894      AACAAGCTTCTTGGGCGGGAAGTGTACAGCTCCCACATGCAGTTGGGTGGTCCTAAGATC  5559
CH05.2958      AACAAGCTTCTTGGGCGGGAAGTGTACAGTTCCCACATGCAGTTGGGTGGTCCTAAGATC  5559
AH09.362       AACAAGCTTCTTGGGCGGGAAGTGTACAGCTCCCACATGCAATTGGGTGGTCCTAAGATA  5559
BH09.424       AACAAGCTTCTTGGGCGGGAAGTGTACAGCTCCCACATGCAATTGGGTGGTCCTAAGATC  7125
CH09.447       AACAAGCTTCTTGGGCGGGAAGTGTACAGCTCTCACATGCAATTGGGTGGTCCTAAGATC  5556
               ***************************** ** ******** ***************** 

BH05.2894      ATGGCAACCAATGGTGTTGTCCACTTGACTGTTACAGATGACCTTGAAGGTGTTTCCAAC  5619
CH05.2958      ATGGCAACCAATGGTGTTGTCCACTTGACTGTTACAGATGACCTTGAAGGTGTTTTCAAC  5619
AH09.362       ATGGCTACCAATGGTGTTGTCCACTTGACTGTTACAGATGACCTTGAAGGTGTTTCCAAT  5619
BH09.424       ATGGCTACCAATGGTGTTGTCCACTTGACTGTTTCAGATGACCTTGAAGGTGTTTCCAAT  7185
CH09.447       ATGGCTACCAATGGTGTTGTCCACTTGACTGTTTCAGATGACCTTGAAGGTGTTTCCAAT  5616
               ***** *************************** ********************* *** 

BH05.2894      ATATTGAGGTGGCTTAGCTATGTTCCTGCCAACATTGGTGGACCTCTTCCTATTACAAAA  5679
CH05.2958      ATATTGAGGTGGCTTAGCTATGTTCCTGCCAACATTGGTGGACCTCTTCCTATTACAAAA  5679
AH09.362       ATATTGAGATGGCTCAGCTATGTTCCTGCGAACATTGGTGGACCTCTTCCTATTACAAAA  5679
BH09.424       ATATTGAGGTGGCTCAGCTATGTTCCTGCTAACATTGGTGGACCTCTTCCTATTACAAAA  7245
CH09.447       ATATTGAGGTGGCTCAGCTATGTTCCTGCTAACATTGGTGGACATCTTCCTATTACAAAA  5676
               ******** ***** ************** ************* ****************

BH05.2894      CCTTTGGACCCACCAGACAGACCTGTTGCATACATCCCTGAGAACACATGTGATCCGCGT  5739
CH05.2958      CCTTTGGACCCACCAGACAGACCTGTTGCATACATCCCTGAGAACACATGTGATCCGCGT  5739
```

```
AH09.362      CCTTTGGACCCACCAGACAGACCTGTTGCATACATCCCTGAGAACACATGTGATCCGCGT  5739
BH09.424      CCTTTGGACCCACCAGACAGACCTGTTGCATACATCCCTGAGAACACATGTGATCCGCGT  7305
CH09.447      CCTTTGGACCCACCAGACAGACCTGTTGCATACATCCCTGAGAACACATGTGATCCGCGT  5736
              ************************************************************

BH05.2894     GCAGCCATTCGTGGTGTAGATGACAGCCAAGGGAAATGGTTGGGTGGTATGTTTGACAAA  5799
CH05.2958     GCAGCTATTCGTGGTGTAGATGACAGCCAAGGGAAATGGTTGGGTGGTATGTTTGACAAA  5799
AH09.362      GCAGCCATTCGTGGTGTAGATGACAGCCAAGGGAAATGGTTGGGTGGTATGTTCGACAAA  5799
BH09.424      GCAGCCATTCGTGGTGTAGATGACAGCCAAGGGAAATGGTTGGGTGGTATGTTCGACAAA  7365
CH09.447      GCAGCCATTCGTGGTGTAGATGACAGCCAAGGGAAATGGTTGGGTGGTATGTTCGACAAA  5796
              ***** *********************************************** ******

BH05.2894     GACAGCTTTGTGGAGACATTTGAAGGATGGGCAAAGACTGTGGTTACTGGCAGAGCAAAG  5859
CH05.2958     GACAGCTTTGTGGAGACATTTGAAGGATGGGCGAAGACTGTGGTTACTGGCAGAGCAAAG  5859
AH09.362      GACAGCTTTGTGGAGACATTTGAAGGATGGGCGAAAACAGTGGTTACTGGCAGAGCAAAG  5859
BH09.424      GACAGCTTTGTGGAGACATTTGAAGGATGGGCGAAAACAGTGGTTACTGGCAGAGCAAAG  7425
CH09.447      GACAGTTTTGTGGAGACATTTGAAGGATGGGCGAAAACAGTGGTTACTGGCAGAGCAAAG  5856
              ***** ************************** ** ** *********************

BH05.2894     CTTGGAGGAATTCCTGTTGGTGTCATAGCTGTGGAGACACAAACCATGATGGAGCTTATC  5919
CH05.2958     CTTGGAGGAATTCCTGTTGGTGTCATAGCTGTGGAGACACAAACCATGATGGAGCTTATC  5919
AH09.362      CTTGGAGGAATCCCTGTCGGTGTCATAGCTGTGGAGACGCAAACAATGATGCAGCTTATC  5919
BH09.424      CTTGGAGGAATCCCTGTCGGTGTCATAGCTGTGGAGACGCAAACAATGATGCAGCTTATC  7485
CH09.447      CTTGGAGGAATCCCTGTTGGTGTCATAGCTGTGGAGACGCAAACAATGATGCAGCTTATC  5916
              *********** ***** ******************** ***** ****** ********

BH05.2894     CCTGCTGATCCAGGCCAGCTTGATTCCCATGAGCGATCTGTTCCTCGGGCTGGACAAGTG  5979
CH05.2958     CCTGCTGATCCAGGCCAGCTTGATTCCCATGAGCGATCTGTTCCTCGGGCTGGACAAGTG  5979
AH09.362      CCTGCTGATCCAGGCCAGCTTGATTCCCATGAGCGATCTGTTCCTCGTGCTGGACAAGTG  5979
BH09.424      CCTGCTGATCCTGGCCAGCTTGATTCCCATGAGCGATCTGTTCCTCGTGCTGGACAAGTG  7545
CH09.447      CCTGCTGATCCAGGCCAGCTTGATTCCCATGAGCGATCTGTTCCTCGTGCTGGACAAGTG  5976
              *********** *********************************** ************

BH05.2894     TGGTTCCCAGATTCTGCAACCAAGACAGCTCGGGCGTTGTTGGATTTCAACCGTGAAGGA  6039
CH05.2958     TGGTTCCCAGATTCTGCAACCAAGACAGCTCGGGCGTTGTTGGATTTCAACCGTGAAGGA  6039
AH09.362      TGGTTCCCAGATTCTGCAACAAAGACAGCTCAGGCATTATTGGACTTCAACCGTGAAGGA  6039
BH09.424      TGGTTCCCAGATTCTGCAACAAAGACAGCTCAGGCATTATTGGACTTCAACCGTGAAGGA  7605
CH09.447      TGGTTCCCAGATTCTGCAACAAAGACAGCTCAGGCATTATTGGACTTCAACCGTGAAGGA  6036
              ******************** ********** *** ** ***** ***************

BH05.2894     TTGCCTCTATTCATCCTTGCTAACTGGAGAGGTTTTTCCGGTGGACAAAGAGATCTGTTT  6099
CH05.2958     TTGCCTCTATTCATCCTTGCTAACTGGAGAGGTTTTTCCGGTGGACAAAGGGATCTGTTT  6099
AH09.362      TTGCCTCTGTTCATCCTTGCTAACTGGAGAGGTTTCTCCGGTGGACAAAGAGATCTGTTT  6099
BH09.424      TTGCCTCTGTTCATCCTTGCTAACTGGAGAGGTTTCTCCGGTGGGCAAAGAGATCTGTTT  7665
CH09.447      TTGCCTCTGTTCATCCTTGCTAACTGGAGAGGTTTCTCCGGTGGACAAAGAGATCTGTTT  6096
              ******** ************************** ******** ***** *********

BH05.2894     GAAGGAATTCTTCAGGCTGGGTCAACAATTGTTGAGAACCTTAGGACATACAATCAGCCT  6159
CH05.2958     GAAGGAATTCTTCAGGCTGGGTCAACAATTGTTGAGAACCTTAGGACATACAATCAGCCT  6159
AH09.362      GAAGGGATTCTTCAGGCTGGGTCAACAATTGTTGAGAATCTTAGGACATATAATCAACCT  6159
BH09.424      GAAGGGATTCTTCAGGCTGGGTCAACAATTGTTGAGAATCTTAGGACATATAATCAACCT  7725
CH09.447      GAAGGGATTCTTCAGGCTGGGTCAACAATTGTTGAGAATCTTAGGACATATAATCAACCT  6156
              ***** ******************************** *********** ***** ***

BH05.2894     GCATTTGTCTACATTCCTATGGCTGGAGAGCTGCGTGGAGGAGCTTGGGTTGTGGTTGAT  6219
CH05.2958     GCATTTGTCTACATTCCTATGGCTGGAGAGCTGCGTGGAGGAGCTTGGGTGGTGGTTGAT  6219
AH09.362      GCATTTGTCTACATTCCTATGGCTGGAGAGCTGCGTGGAGGAGCTTGGGTTGTGGTTGAT  6219
BH09.424      GCATTTGTCTACATTCCTATGGCTGGAGAGCTGCGTGGAGGAGCTTGGGTTGTGGTTGAT  7785
CH09.447      GCATTTGTCTACATTCCTATGGCTGGAGAGCTGCGTGGAGGAGCTTGGGTTGTGGTTGAT  6216
              ************************************************** *********
```

```
BH05.2894      AGCAAGATAAATCCAGACCGCATTGAGTGTTATGCTGAGAGGACTGCTAAAGGAAATGTT 6279
CH05.2958      AGCAAAATAAATCCAGACCGCATAGAGTGTTATGCTGAGAGGACTGCTAAAGGAAATGTT 6279
AH09.362       AGCAAAATAAATCCAGACCGAATTGAGTGTTATGCTGAGAGGACTGCGAAAGGCAATGTT 6279
BH09.424       AGCAAAATAAATCCAGACCGAATTGAGTGTTATGCTGAGAGGACTGCGAAAGGCAATGTT 7845
CH09.447       AGCAAAATAAATCCAGACCGAATTGAGTGTTATGCTGAGAGGACTGCGAAAGGCAATGTT 6276
               ***** ************** ** *********************** ***** ******

BH05.2894      CTTGAACCTCAAGGGTTAATTGAAATCAAGTTCAGGTCAGAGGAACTCCAAGACTGTATG 6339
CH05.2958      CTTGAACCTCAAGGGTTAATTGAAATCAAGTTCAGGTCAGAGGAACTCCAAGACTGTATG 6339
AH09.362       CTTGAACCTCAAGGGTTAATTGAAATCAAGTTCAGGTCAGAGGAACTCCAAGACTGTATG 6339
BH09.424       CTTGAACCTCAAGGGTTAATTGAAATCAAGTTCAGGTCAGAGGAACTCCAAGACTGTATG 7905
CH09.447       CTTGAACCTCAAGGATTAATTGAAATCAAGTTCAGGTCGGAGGAACTCCAAGACTGTATG 6336
               ************** *********************** *********************

BH05.2894      GGTAGGCTTGATCCAGAGTTGATAAATCTGAAAGCAAAACTCCAAGGTGCAAAGCTTGGA 6399
CH05.2958      GGTAGGCTTGATCCAGAGTTGATAAATCTGAAAACAAAACTCCAAGGTGCAAAGCTTGGA 6399
AH09.362       GGTAGGCTTGACCCAGAGTTGATAAATATGAAAGCAAAACTCCAAGGTGCAAAGGTTGGA 6399
BH09.424       GGTAGGCTTGACCCAGAGTTGATAAATCTGAAAGCAAAACTCCAAGGTGCAAAGGTTGGA 7965
CH09.447       GGTAGGCTTGACCCAGAGTTGATAAATCTGAAAGCAAAACTCCAAGGTGCAAAGGTTGGA 6396
               *********** *************** ****** ******************* *****

BH05.2894      AATGGAAGTTTACCTGACATGGAATCCATTCAGAAGAGTATAGAAGCTCGTACAAAACAG 6459
CH05.2958      AATGGAAGTCTACCTGACATGGAATCCATTCAGAAGAGTATAGAAGCTCGTACAAAACAG 6459
AH09.362       AATGGAAGCCTACCTGACATAGAATCCCTTCAGAAGAGTATAGAAGCTCGTACCAAACAA 6459
BH09.424       AATGGAAGCCTACCTGACATAGAATCCCTTCAGAAGAGTATAGAAGCTCGTACCAAACAA 8025
CH09.447       AATGGAAGCATACCTGACATAGAATCCCTTCAGAAGAGTATAGAAGCGCGTACCAAACAA 6456
               ********  ********** ****** ******************* ***** ***** 
```

Suppl. Fig. S1. Partial *ACCase* genes from all three *E. crus-galli* subgenomes. Alignment of the partial *ACCase* genes showing primers targeting (Yellow: ACCase1F– ACCase1R; Light blue: ACCase3F – ACCase3R; Green: ACCase7F – ACCase7R). Red indicates the position where mutations conferring herbicide resistance are found. Asterisk (*) indicates the same nucleotide in all three subgenomes. Absence of * indicates there is at least one different nucleotide between them.

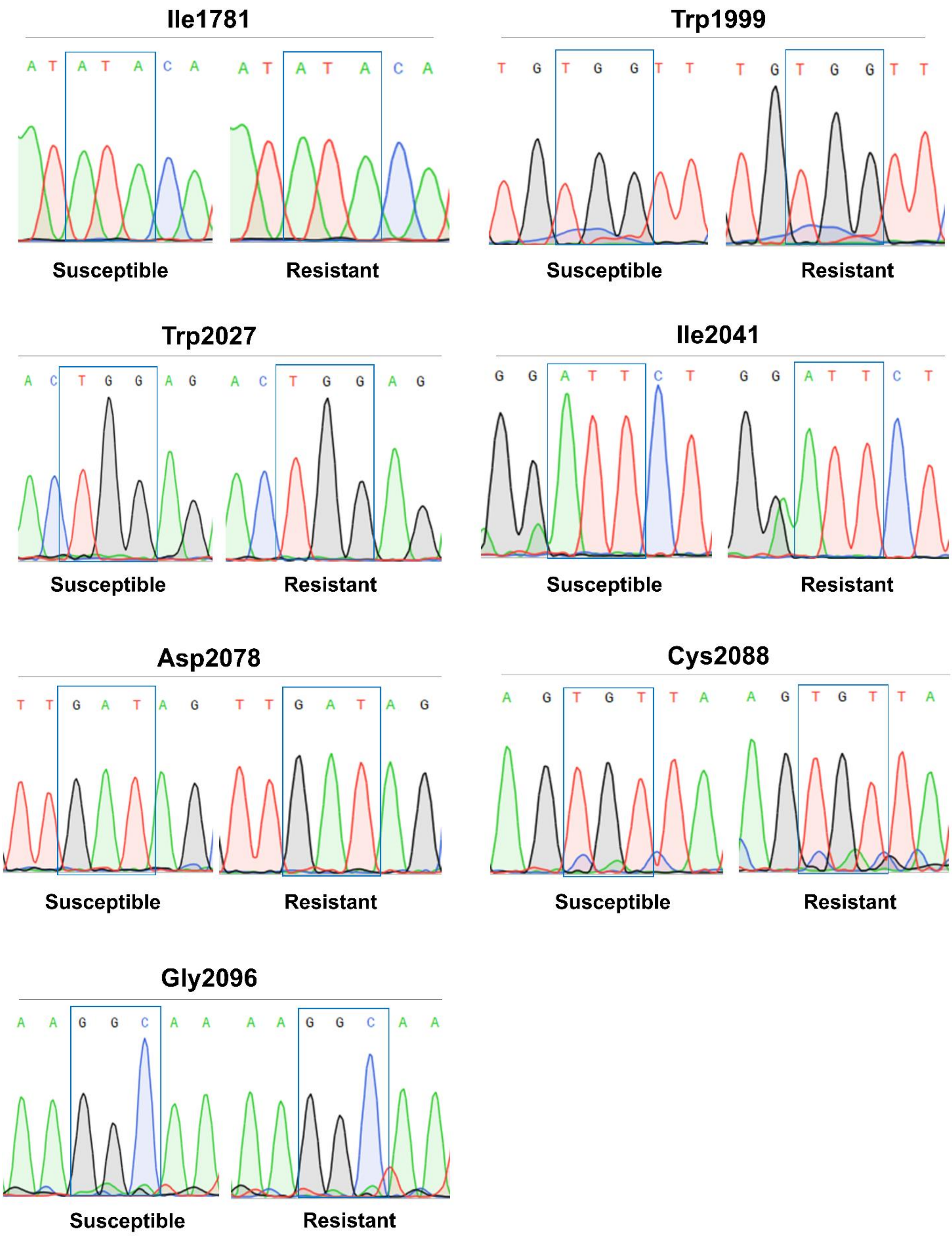

Suppl. Fig. S2. Sequencing of *ACCase* genes in cyhalofop-butyl susceptible and resistant biotypes. The highlighted positions are known to confer herbicide resistance when amino acid substitutions occur.

Supplementary Table

Suppl. Table S1. LC-MS/MS settings utilized to detect cyhalofop-butyl and cyhalofop acid

Compounds	Mode	MRM	Dwell (ms)	Q1 (V)	CE (V)	Q3 (V)
Cyhalofop-butyl	+	358.0>256.0	100	-17	-15	-27
	+	358.0>184.1	100	-10	-46	-21
Cyhalofop acid	-	300.2>228.1	100	13	11	24
	-	300.2>208.0	100	19	19	21
	-	300.2>100.1	100	19	47	17
